# Point-of-Care Testing for Multiple Cardiac Markers Based on a Snail-Shaped Microfluidic Chip

**DOI:** 10.3389/fchem.2021.741058

**Published:** 2021-10-04

**Authors:** Binfeng Yin, Xinhua Wan, Changcheng Qian, A. S. M. Muhtasim Fuad Sohan, Songbai Wang, Teng Zhou

**Affiliations:** ^1^ School of Mechanical Engineering, Yangzhou University, Yangzhou, China; ^2^ School of Chemistry and Chemical Engineering, Shanxi University, Taiyuan, China; ^3^ Mechanical and Electrical Engineering College, Hainan University, Haikou, China

**Keywords:** cardiac markers, snail-shaped microfluidic chip, POCT, chemiluminescence, multiplex detection

## Abstract

Existing methods for detecting cardiac markers are difficult to be applied in point-of-care testing (POCT) due to complex operation, long time consumption, and low sensitivity. Here, we report a snail-shaped microfluidic chip (SMC) for the multiplex detection of cTnI, CK-MB, and Myo with high sensitivity and a short detection time. The SMC consists of a sandwich structure: a channel layer with a mixer and reaction zone, a reaction layer coated with capture antibodies, and a base layer. The opening or closing of the microchannels is realized by controlling the downward movement of the press-type mechanical valve. The chemiluminescence method was used as a signal readout, and the experimental conditions were optimized. SMC could detect cTnI, CK-MB, and Myo at concentrations as low as 1.02, 1.37, and 4.15. The SMC will be a promising platform for a simultaneous determination of multianalytes and shows a potential application in POCT.

## 1 Introduction

Point-of-care testing (POCT) is a significant development direction of medical examination, providing test results nearby and quickly and guiding diagnosis and treatment (Dincer et al., 2017; Yin et al., 2017; Chen et al., 2018; Liu et al., 2019). Especially for acute diseases, early self-screening using the POCT technology in ambulances, communities, and homes is a powerful means of ensuring a long and healthy life. Acute myocardial infarction (AMI) is a common disease that seriously endangers human life. The mortality rate of AMI maintains at a high level due to acute onset and risky processes. Patients often lost their lives within a short period after a heart attack. The golden treatment time is only 3 h, and the mortality rate will increase by 12% if the patient is treated 6 h after onset (McCord et al., 2001). Hence, it is an urgent need for prompt diagnosis and timely intervention of AMI to save the lives of cardiac patients. Existing diagnostic methods are combined with the symptoms and electrocardiogram (ECG) ST-segment elevation (Hofmann et al., 2017). Chest pain is the most common symptom of AMI and may be accompanied by sweating, nausea or vomiting, and fainting. Patients always have individual differences, and this is difficult to quantify the symptoms. According to a report, 15–40 percent of AMI can happen with no typical cardiac symptoms (Davis et al., 2004). It means that the symptoms of the patients are not a definite standard for the diagnosis of AMI. Since ST-segment deviation could occur in other diseases, including acute pericarditis, left ventricular hypertrophy, and left bundle branch block, the ECG auxiliary examination cannot accurately diagnose AMI in some situations. Furthermore, non-ST-segment elevation myocardial infarction, unstable angina pectoris, and some super early-stage AMI onsets do not display an ECG ST deviation (Thygesen et al., 2012; Ibanez et al., 2018). In addition, an ECG examination has high requirements for equipment, which is challenging to meet the needs of POCT. Therefore, it is of great significance to detect cardiac markers with high sensitivity and specificity for early diagnosis of AMI (Lim et al., 2020).

Common cardiac markers include creatine kinase-MB (CK-MB), myoglobin (Myo), and cardiac troponin I (cTnI). CK-MB is mostly expressed in cardiomyocytes and released 3 h after the onset of AMI, with a peak at about 24 h. The blood level of CK-MB is used to assist in diagnoses of AMI (Holmvang et al., 2002; Landesberg et al., 2003). Once the cardiomyocyte is injured after 1 h of the AMI onset, Myo is released into the blood circulation quickly and peaks after 6 h. Thus, Myo is used as a sensitive indicator for the early diagnosis of AMI (Pu et al., 2017; Ji et al., 2019). cTnI is specifically expressed in myocardium cells and is the gold standard for the diagnosis of myocardium necrosis. The content of cTnI elevated in serum reflects the damage of cardiomyocytes. The occurrence time of cTnI in serum is similar to that of CK-MB, but it can persist for about 1 week (Ohman et al., 1996; Keller et al., 2009). In normal human serum, the content of CK-MB is 0.3–4.0 ng/ml, the content of Myo is 12–75 ng/ml, and the content of cTnI is less than 0.2 ng/ml. Due to the low level of cTnI in serum, it is difficult to be accurately detected. The earliest occurrence time of Myo, the high specificity of cTnI, and the prognostic value of CK-MB can cover the whole process of AMI. Multiplex detection of CK-MB, Myo, and cTnI is helpful for the early and rapid diagnosis of AMI, disease grade assessment, and evaluation of therapeutic effects (Kim et al., 2010). It is particularly important to develop multiplex detecting techniques of cardiac markers suitable for POCT to save the lives of cardiac patients.

Microfluidic chips can be applied to biochemical analysis in a confined microchannel. It possesses many advantages appropriate for POCT (Dong et al., 2019; Hiramoto et al., 2019; Yin et al., 2021), such as rapid reaction, low reagent consumption, a high throughput, low cost, and high automation. Chemiluminescence (CL) is the emission of luminescence resulting from a chemical reaction between labeled enzymes and the substrate. The content of substances is proportional to the intensity of the luminous signal. It can realize sensitive detecting of trace markers in samples and be used as a conventional detector in microfluidic chips (Hu et al., 2017; Soares et al., 2017; Xie et al., 2019). A biochip with 3 × 8 wells array was constructed to simultaneously detect three cardiac markers cTnI, CK-MB, and Myo (Zhao et al., 2019). However, it depends on medical testing equipment and consumes time more than 1 h. The design of patterned chips can avoid complicated pretreatment steps and shorten the immunoassay process. Combing CL means and microfluidic chip platforms can meet the requirement of POCT for multiple cardiac markers (Yang et al., 2020).

Herein, we report a snail-shaped microfluidic chip (SMC) using a CL detector for multiplex detection of CK-MB, Myo, and cTnI. We demonstrate this technique by fabricating a simple microchip with a sandwich structure. It consists of two polydimethylsiloxane (PDMS) layers with microchannels outside and a silicon film coated with capture antibodies in the middle. With this simple, miniaturized sandwich chip, we can detect CK-MB, Myo, and cTnI simultaneously in serum by scanning the detection zone with a portable CL image analysis system. By analyzing the CL signals, we can determine the concentrations of cardiac markers rapidly and accurately. This approach will be particularly applicable for detecting cardiac markers at the early screening in emergency vehicles, communities, and families where advanced laboratory facilities are not accessible.

## 2 Experimental Section

### 2.1 Materials and Instruments

Capture antibodies of CK-MB (CK-MB-Ab_1_), Myo (Myo-Ab_1_), and cTnI (cTnI-Ab_1_) are from Abcam (United Kingdom). The CK-MB protein is from EastCoast Bio (United States). The Myo protein and recombinant human cardiac troponin I protein are from Abcam (United Kingdom). Detection antibodies of CK-MB (CK-MB-Ab_2_), Myo (Myo-Ab_2_), and cTnI (cTnI-Ab_2_) conjugated with HRP are from Abcam (United Kingdom). Supersensitive chemiluminescent substrate reagent kits are from Beijing Labgic Technology Co., Ltd. (Beijing, China). Bovine serum albumin fraction V (BSA) powder is from Tianjin Kangyuan Biotechnology Co., Ltd. (Tianjin, China). Phosphate-buffered saline (PBS) tablets and Tween-20 are from Amresco (United States), which are used to prepare 0.01 mol/L PBS solution (pH 7.4) and 0.5% PBST solution. The capture antibodies and antigens are diluted in PBS solution. The detection antibodies are diluted in 0.05% BSA solution. Polydimethylsiloxane (PDMS) and a curing agent (Sylgard 184) from Dow Corning Inc. (United States) are used to prepare the microfluidic chips. A 500*500*0.1 mm silicon film used to coat the capture antibody is from Shanghai Shentong Rubber and Plastic Products Co., Ltd. (Shanghai, China).

The CL image analysis system is purchased from BIO-OI Co., Ltd. (Guangzhou, China). A PT-10s Plasma Cleaner is produced by SANHOPTT Co., Ltd. (Shenzhen, China). A 202-00T electric thermostatic drying oven is purchased from LICHEN-BX Co., Ltd. (Shanghai, China). A DZF-6020A vacuum drying oven is purchased from LICHEN-BX Co., Ltd. (Shanghai, China). An F900 industrialization FDM printer is produced by Stratasys Ltd. (United States). A Lite 600HD 3D printer is produced from SHINING 3D Technology Co., Ltd. (Yangzhou, China). An IMM 3000 metallographic microscopy system is purchased from MEGA Instruments Co., Ltd. (Suzhou, China).

### 2.2 Design of the Snail-Shaped Microfluidic Chip

**FIGURE 1:**
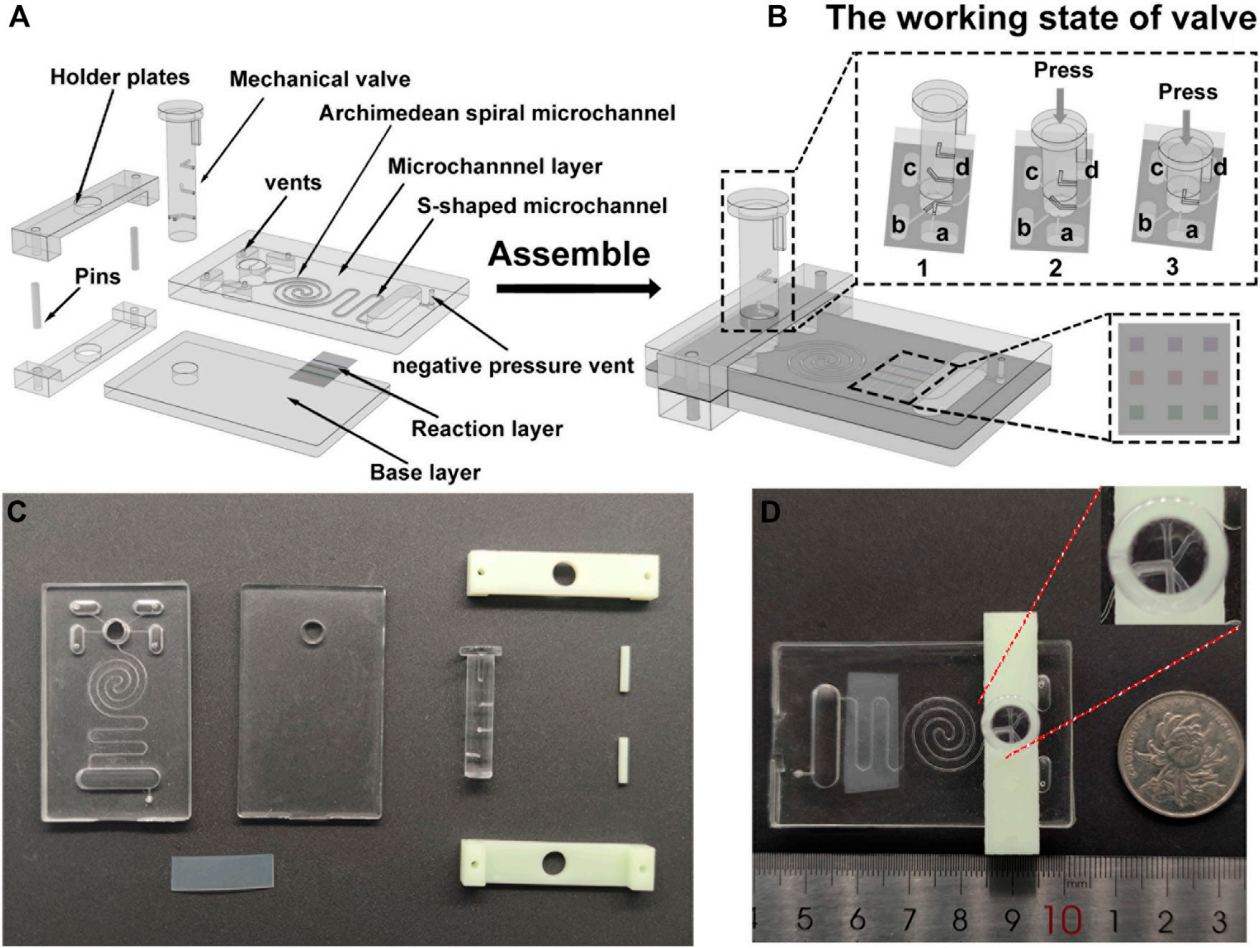
Design and assembly of an integrated SMC with the PM valve for the chemiluminescence immunoassay. **(A)** Schematic diagram of the top microchannel layer with embedded microchannels, the middle reaction layer with patterned antibody stripes, the base layer, and the PM valve before assembly. **(B)** Schematic diagram of the integrated chip and the operation manual of the PM valve. The inset figure is the enlargement of the side view for the PM valve connected to the reservoirs and nine reaction spots on the silicon film. **(C)** Picture of the SMC mold and PM valve. **(D)** Image of the assembled SMC.

We designed the three-dimensional model of the SMC using SolidWorks^®^ 2016 software. The SMC (35 mm long, 57 mm wide) for the chemiluminescent reaction consists of three layers: a microchannel layer (4 mm thick) on top, a reaction layer (silicon film, 0.1 mm thick) in the middle, and a base layer (3 mm thick) at the bottom (Figure 1A).

The top microchannel layer comprises four capsule-shaped reservoirs (7 mm long, 3 mm wide, 3 mm deep, and with a capacity of 60 μl) for depositing samples, detecting antibodies, PBST, and Luminol-H_2_O_2_ CL substrates, respectively. To load reagent samples conveniently and maintain the pressure balance of the chip, a vent (1.0 mm in diameter) is drilled by a puncher on the top of the reservoir. To mix samples efficiently, an Archimedean spiral mixer is designed. At downstream of the Archimedean spiral microchannel, an S-shaped channel is devised. The sizes of Archimedean spiral and S-shaped microchannels are both 400 μm wide and 400 μm deep with a total capacity of 30 μl. All reservoirs converge into the Archimedean spiral microchannel and S-shaped microchannel through a press-type mechanical (PM) valve. The end of the S-shaped channel is a waste chamber. A negative pressure vent (a through hole with a diameter of 1.0 mm) in the waste chamber is used to connect a pump for fluid driving. The middle reaction layer is a silicon film patterned with three different capture antibody strips in advance (Supplementary Figure S1). After the fluid runs across the S-shaped microchannel on the surface of the reaction layer, a microarray zone with nine spots for multianalyte detection is formed by CL reaction (Figure 1B). The bottom base layer has the same size as the top microchannel layer, and they both contain a hole in the top to insert the PM valve.

The PM valve is used to connect different reservoirs with the Archimedean spiral microchannel. Each reagent in different reservoirs is injected into the microchannel of the chip by pressing the PM valve to a proper height which opens a peristaltic pump. To ensure the stability of the movement of the PM valve, we designed an external holder composed of two holder plates (a thickness of 2 mm) with a hole in the middle for inserting the valve and two pin holes in both ends for inserting pins to immobilize the chip (Figure 1B).

### 2.3 Fabrication and Assembly of the Snail-Shaped Microfluidic Chip

First, we select a 3D printer with a resin to produce the mold of the microchannel layer, base layer, PM valve, and external holder (Figures 1A,C). The material used for the mold should be easy to process and shape. In addition, it should be resistant to high temperature to avoid deformation in the oven and ensure that the size of the microchannel is uniform. We remove the unformed material from the surface via an ultrasonic machine and remove surface burrs by sanding. Then, the PDMS matrix and curing agent mixture are put into a vacuum drying oven to remove the bubbles. The bubble-free mixture is poured into the mold, and the tiny bubbles on the surface are blown away with airflow. Setting the temperature at 85°C for 30 min in a thermostatic oven, the formed microchannel and base layers are removed from the mold with tweezers. After removing the dust on the surface, a hole with a diameter of 1 mm is punched on the top of the reservoir. Under the condition of a 200 W power and 1.5 L/min oxygen flow, the microchannel and base layers are bombarded with plasma for 60 s. Hence, the hydrophobic surface was changed into a hydrophilic surface for the breakage of the silicon–oxygen bond.

After that, we place the silicon film between the microchannel layer and the base layer. Let the antibody strips of the silicon film intersect the S-channel of the top layer. The bubbles in the double PDMS layers were squeezed out. They were made tightly bonded using a plasma cleaner. Because the holder plates shade the reservoirs (Figure 1D), the detection antibody solution and CL substrate are added into the lower two reservoirs (Figure 1Ba,d) in advance. One holder plate is placed at the bottom of the double PDMS layer, and another one is installed at the top. Two short pins are inserted into pin holes on both ends of the holders to fasten the two holders. Last, the PM valve was loaded into the through hole, and the assembly of the chip is achieved (Figures 1B,D).

### 2.4 Pretreatment of the Snail-Shaped Microfluidic Chip

A PDMS microfluidic chip with three straight channels (500 μm wide and 300 μm deep) arranged in parallel with a spacing of 3 mm is vertically placed on the surface of the silicon film. CK-MB-Ab_1_, Myo-Ab_1_, and cTnI-Ab_1_ are injected into the channels. After 1 h, the silicon film is cleaned with PBST buffer three times to remove the uncoated capture antibodies (Supplementary Figure S1). Then, the silicon film is assembled into the SMC. 50 μl of 5% BSA solution is added to any reservoir after SMC is assembled, and the PM valve is pressed down to the corresponding height. A negative pressure peristaltic pump is used to pump 5% BSA solution into the main microchannel until it is filled with 5% BSA solution. 5% BSA solution is incubated for 15 min to block the vacant sites on the silicon film and the microchannel to reduce nonspecific adsorption of proteins.

## 3 Results and Discussion

### 3.1 Principle and Characterization

**FIGURE 2:**
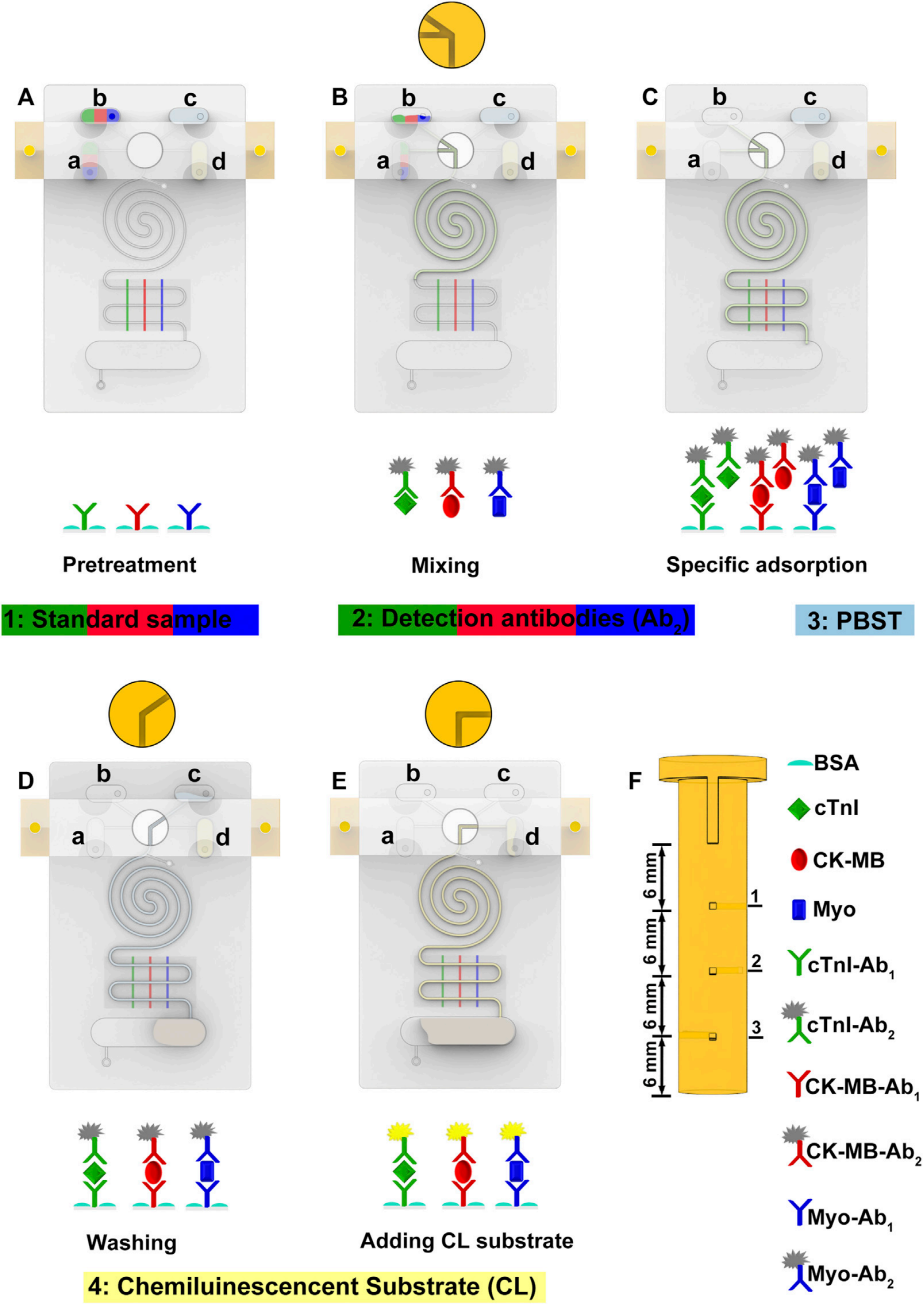
Validation of the precise microchannel and through hole of the PM valve. **(A)** Longitudinal section of the S-shaped microchannel. The designed width is 400 μm, the actual width is 399.49 μm, and the deviation is 0.13%. **(B)** Longitudinal section of the Archimedean spiral microchannel. The design width is 400 μm, the actual width is 405.10 μm, and the deviation is 1.28%. **(C)** Cross-section of the S-shaped microchannel. The designed height is 400 μm, the actual height is 400.34 μm, and the deviation is 0.09%. **(D)** Cross-section of the PM valve. The designed width is 400 μm; the actual width is 391.09, 403.01, and 409.04 μm; and the average deviation is 1.75%.

Fluid flow in microfluidic chips is a crucial factor for biochemical analysis (Terray et al., 2002). We employ a PM valve to switch reservoirs to the microchannel and a negative pressure system to control fluid flow. The PM valve comprises three group holes in different directions. Each group hole is 6 mm apart in an altitude interval (Figure 2F). When the hole of the PM valve connects to the reservoir, the liquid can be smoothly pumped into the main channel by the negative pressure system. When the nonhole part of the PM valve connects to the reservoir, the liquid is blocked and will not flow into the main channel. To characterize the fluid flow, we use COMSOL software to simulate the flow velocity in the microchannel (Supplementary Figure S2). Under a negative pressure of 3.5 kPa, the flow rate is stabilized.

As a mixing vessel, the Archimedean spiral microchannel can make samples, and detection antibodies released from different reservoirs mix efficiently and quickly (Viktorov et al., 2016; Lee and Fu, 2018; Raza et al., 2018) and then stimulate an immune response instead of affecting the reaction between the antigen and the capture antibody in the sample (Yin et al., 2016). To assess the blending effect of the Archimedean spiral microchannel in the SMC, we use COMSOL software to simulate the mixing effect of its 3D model under a steady state (Supplementary Figure S3A). The concentration of the two solutions at the inlet is set at 0 and 1 mol/L (Supplementary Figure S3B). The mixing efficiency at the outlet of the Archimedean spiral microchannel is 0.9999 (Supplementary Figure S3C), which can meet the mixing requirements of the next experiment.

**FIGURE 3:**
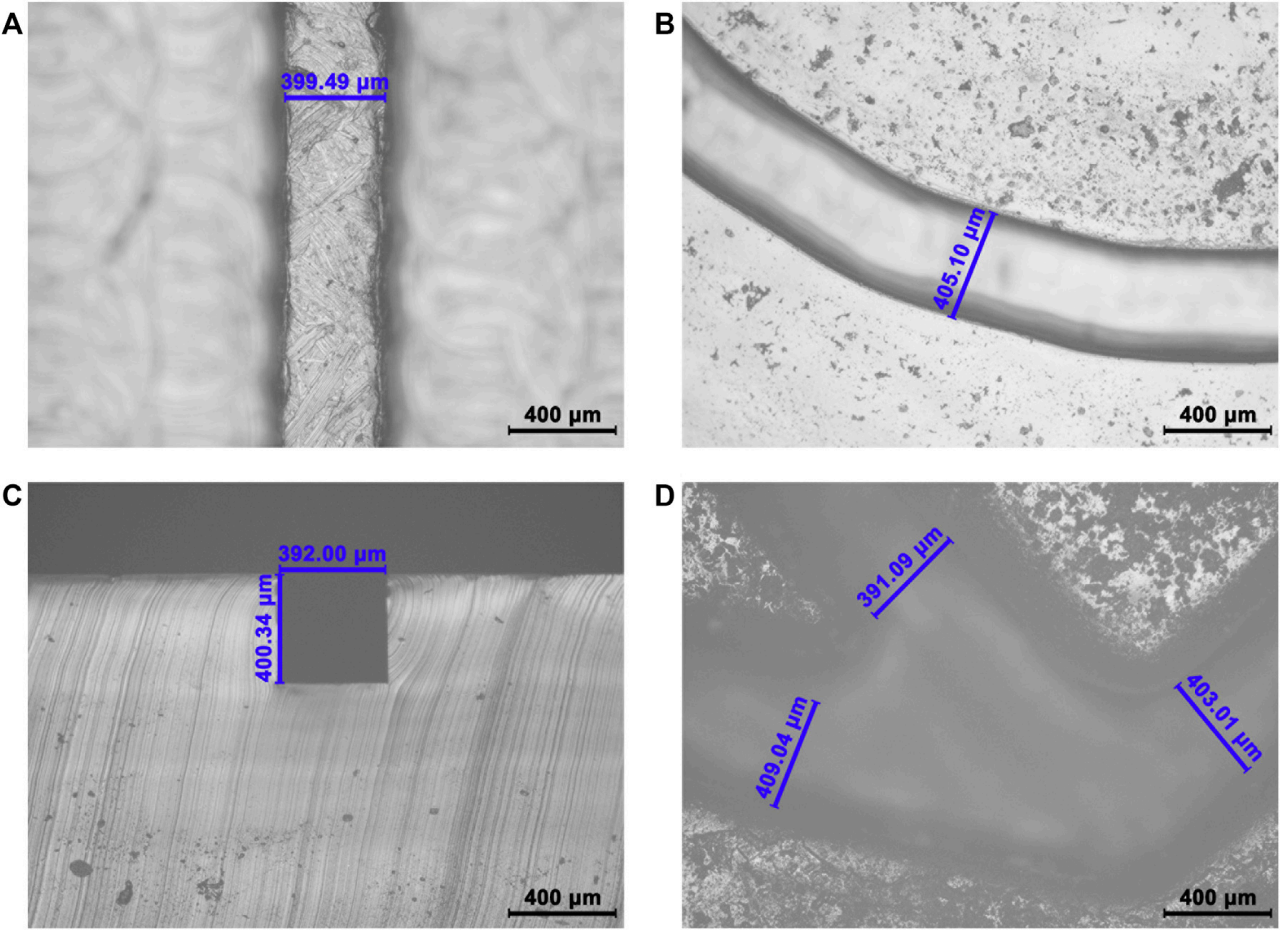
Schematic diagram of detecting samples in SMC. **(A)** Pretreatment of SMC including capture antibody coating, surface blocking, and reagent loading. **(B)** Samples and detection antibodies are mixed and reacted in an Archimedean spiral channel. **(C)** Specific immunoreaction happens in the S-shaped microchannel. **(D)** Washing the unreacted reagent. **(E)** Chemiluminescent substrates are added to produce chemiluminescent signals. **(F)** Schematic diagram of the PM valve.

Compared with lithography and other methods, 3D printing has a simpler process and a lower cost (Chan et al., 2017). It can solve the problems of a small size, high precision, and a complex shape of microfluidic chip channels. Its greatest advantage lies in the low production threshold; convenient, personalized design; and easy manufacture. In order to verify the reliability of this manufacturing method, we measured the deviation between the actual size and the designed size of the S-shaped microchannel (Figure 3A), the Archimedean spiral microchannel (Figures 3B,C), and the PM valve (Figure 3D). The deviations are all below 2.0%. The feasibility of this method to manufacture the precise product is verified. The uniform shape of the microchannel makes the fluid move smoothly in the microchannel, which is the prerequisite and guarantee of an automatic operation. The precise size is also beneficial to the reproducibility and stability of experimental results. SMC with high manufacturing accuracy lays a foundation for batch production in POCT.

### 3.2 Optimization of Coating Capture Antibodies

To obtain an obvious signal, we optimize the concentration of capture antibodies on the silicon film. Silicon films are coated with different concentrations of capture antibodies (3.125, 6.25, 12.5, 25, 50, and 60 μg/ml cTnI-Ab_1_; 5, 10, 20, 40, and 60 μg/ml CK-MB-Ab_1_ and Myo-Ab_1_). We found that the CL signal value of the sample increases upward at first and then gradually becomes a plateau with the increase of the concentration of the capture antibody when cTnI, CK-MB, and Myo are more than 25, 20, and 40 μg/ml, respectively (Supplementary Figure S4). The CL signal values of cTnI, CK-MB, and Myo show a downward trend when their capture antibody concentration is above 50, 40, and 40 μg/ml, respectively. This phenomenon might be caused by the steric hindrance effect, which decreases the sensitivity. Given both cost and sensitivity, 25 μg/ml cTnI-Ab_1_, 20 μg/ml CK-MB-Ab_1_, and 40 μg/ml Myo-Ab_1_ are selected as the concentrations of capture antibodies.

### 3.3 Detecting Process

30 μl of the detection antibody solution (10 μl of 7.2 μg/ml cTnI-Ab_2_, 10 μl of 6 μg/ml CK-MB-Ab_2_, and 10 μl of 9 μg/ml Myo-Ab_2_) and 35 μl of the CL substrate are added into the lower two reservoirs (Figure 1Ba,d) before the assembly of the holder plates. After the chip is assembled, 30 μl of the sample (cTnI, CK-MB, and Myo, 10 μl each) and 50 μl of PBST are added into the upper two reservoirs (Figure 1Bb,c). The peristaltic pump is connected to the chip interface through a vent. The detecting process can be divided into the following steps: 1) push down the PM valve to the first stage, and let reservoir a (detection antibodies) and reservoir b (sample) connect to the main channel (Figure 1B). The peristaltic pump is opened and creates a negative pressure. The sample and detection antibody solution are simultaneously pumped into the Archimedean spiral microchannel and mixed thoroughly. Then, the solution flows into the S-shaped microchannel after the reaction (Figure 2B). 2) Turn off the peristaltic pump and incubate for 15 min. Let the mixed sample react with the capture antibodies on the surface of the silicon film sufficiently. Open the peristaltic pump and pump all the liquid into the waste chamber (Figure 2C). 3) Press down the PM valve to the second stage (Figure 1B), let PBST (reservoir c) go into the main channel for about 15 s of continuous washing until all PBST are pumped into the waste chamber (Figure 2D). 4) Press down the PM valve to the third stage (Figure 1B), and let the CL substrate (reservoir d) flow into the S-shaped microchannel, react with the HRP conjugated on the detection antibodies for 5 s, and then flow into the waste chamber (Figure 2E). A CL image analysis system is used to record the CL signal. Three S-shaped microchannels intersect with three capture antibody strips on the silicon film, yielding a total of nine (3*3) detection spots (Figure 1B). GEL-PRO Analyzer software is used for image processing to obtain the CL values of each spot. Their average CL value is subtracted from the background value to calculate the sample concentration.

### 3.4 Detecting Performance of cTnI, CK-MB, and Myo

**FIGURE 4:**
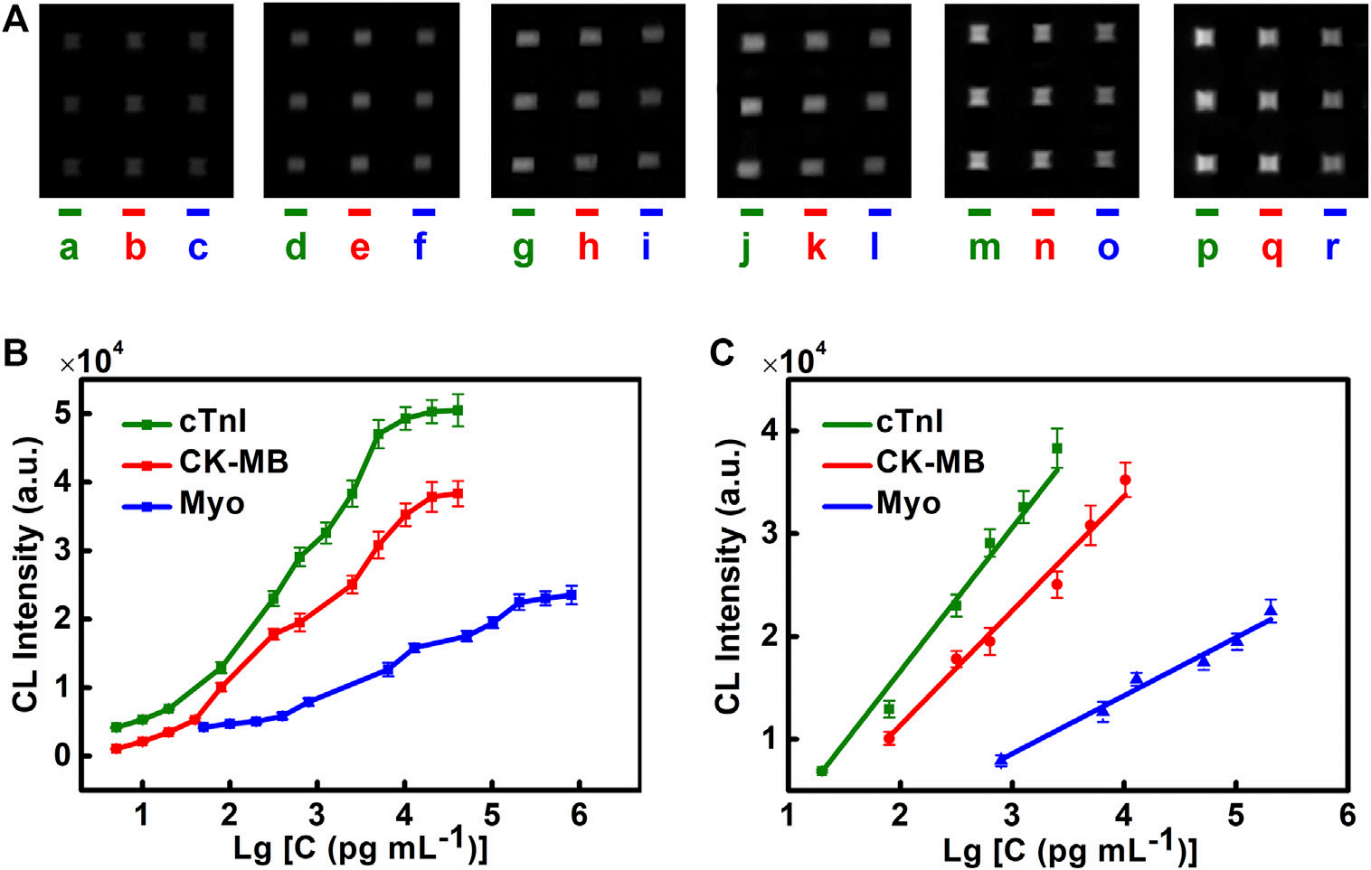
Performance of the microfluidic chemiluminescence immunoassay for simultaneous detection of cTnI, CK-MB, and Myo. **(A)** CL images of cTnI **(left)**, CK-MB **(middle)**, and Myo **(right)** in each chip at different concentrations. The concentrations of cTnI from **left** to **right** are 20, 80, 320, 640, 1,280, and 2,560 pg/ml (lines a, d, g, j, m, and p). The concentrations of CK-MB from **left** to **right** are 0.08, 0.32, 0.64, 2.56, 5.12, and 10.24 ng/ml (lines b, e, h, k, n, and q). The concentrations of Myo from **left** to **right** are 0.8, 6.4, 12.8, 51.2, 102.4, and 204.8 ng/ml (lines c, f, i, l, o, and r). **(B)** Detection curves of cTnI, CK-MB, and Myo. **(C)** Linear range for cTnI, CK-MB, and Myo. The error bar indicates the standard deviation of the CL signals from nine spots in one microfluidic chip.

The detecting performance of the chip is evaluated by the linear range and the limit of detection (LOD). Different concentrations of cTnI, CK-MB, and Myo are injected into the microfluidic chip, and the luminescence intensities are recorded. Detection curves, LOD, and the linear range of different cardiac biomarkers are obtained (Figures 4A–C). We find that cTnI has an ideal linear relationship in the range of 20–2,560 pg/ml ( Y=−11305.16+13968.37X ; $R^{2}=0.98713$ ). CK-MB has an ideal linear relationship in the range of 0.08–10.24 ng/ml ( Y=−10969.63+11160.15X ; $R^{2}=0.98242$ ). Myo has an ideal linear relationship in the range of 0.8–204.8 ng/ml ( Y=−8378.74+5656.95X ; $\;R^{2}=0.97872$ ) (Figure 4C).

LOD is determined by 3 times the standard deviation of the blank. The LODs of cTnI, CK-MB, and Myo are 1.02, 1.37, and 4.15 pg/ml, respectively. They are far below the clinical diagnostic threshold (CK-MB 0.3–4.0 ng/ml, Myo 12–75 ng/ml, cTnI < 0.2 ng/ml). This method shows high sensitivity in detecting cardiac markers and can meet the needs of clinical diagnosis.

### 3.5 Reproducibility of the Chip Detection and Storage Stability

**FIGURE 5:**
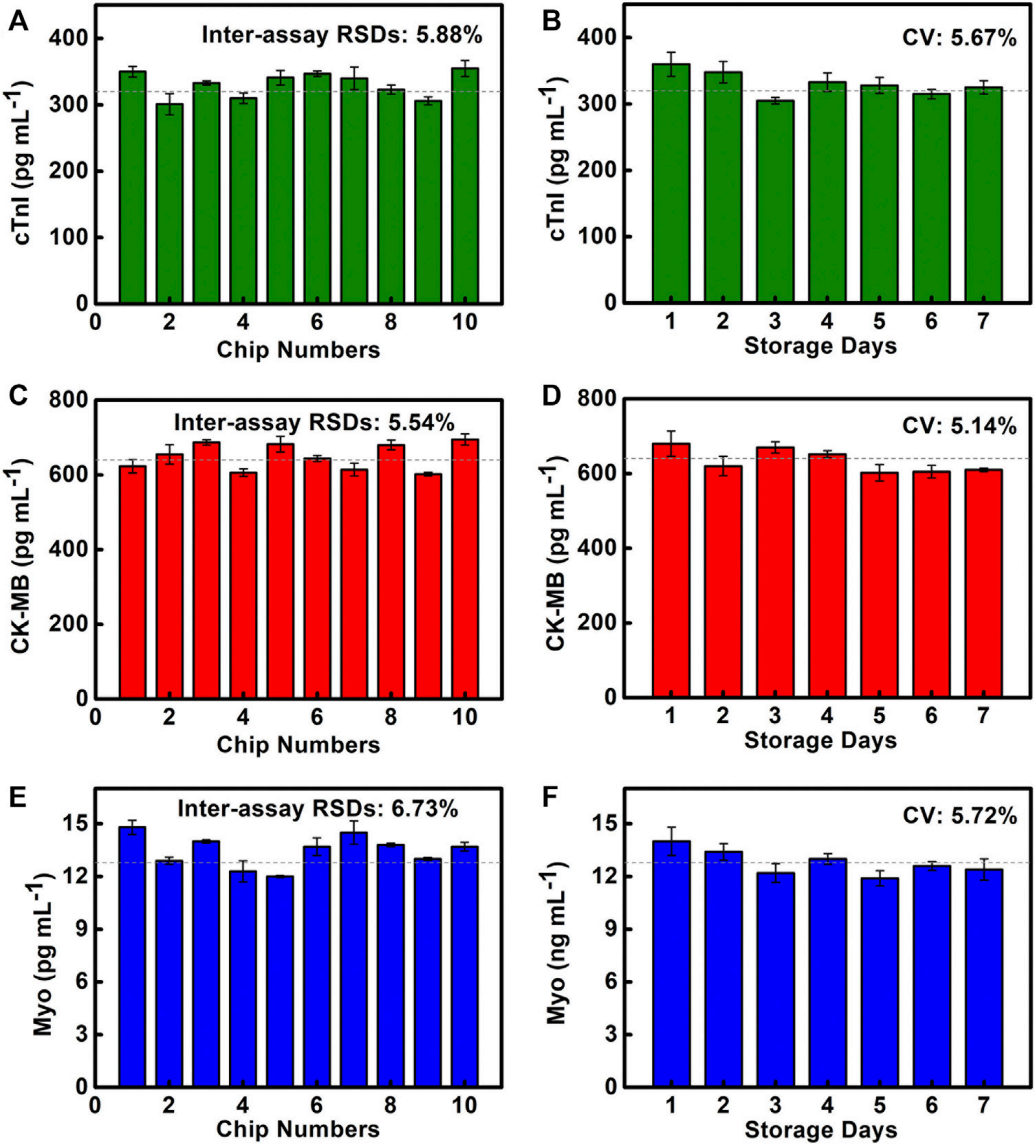
Reproducibility and storage stability of SMC. **(A–C)** Quantitative results of cTnI, CK-MB, and Myo are detected on 10 SMCs. **(D–F)** Quantitative results of cTnI, CK-MB, and Myo are detected on SMC stored for 1–7 days.

To evaluate the reproducibility of the chip detection, 10 chips are used to detect cTnI at 320 pg/ml, CK-MB at 640 pg/ml, and Myo at 12.8 ng/ml, which are selected in the linear range (Figures 5A,C,E). It is discovered that the inter-assay RSDs of cTnI, CK-MB, and Myo are 5.88, 5.54, and 6.73%, respectively, and the inter-assay RSDs are all less than 15%. It shows good reproducibility. This is one of the benefits of using a reliable manufacturing technique, where the precise size and stable shape of SMC allow reliable results to be obtained.

The storage stability of the chip at room temperature (25°C, 1 atm) will directly affect the chip’s performance. We test cTnI of 320 pg/ml, CK-MB of 640 pg/ml, and Myo of 12.8 ng/ml to evaluate the storage stability of SMC, which are stored for 1–7 days (Figures 5B,D,F). The results show that the coefficient of variation (CV) of cTnI, CK-MB, and Myo are 5.67, 5.14, and 5.72%, respectively, indicating that the coated capture antibodies and the preloaded reagents all maintain good activity. The chip has good storage stability, which provides a powerful guarantee for POCT applications.

### 3.6 Clinical Sample Testing

**FIGURE 6:**
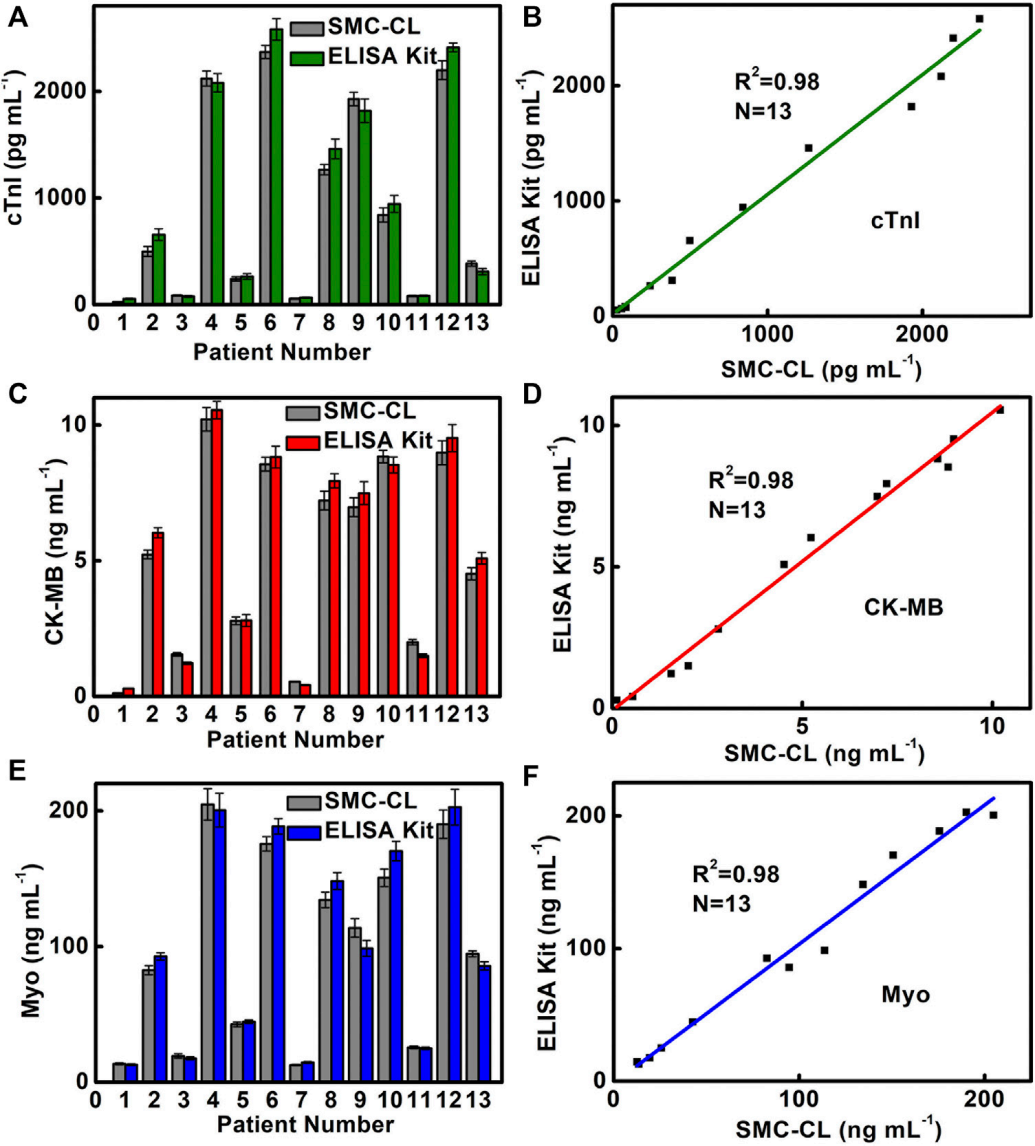
Comparison of **(A)** cTnI, **(C)** CK-MB, and **(E)** Myo detection results and correlation analysis of **(B)** cTnI, **(D)** CK-MB, and **(F)** Myo in clinical samples by SMC and the commercial ELISA Kit. Sample Nos. 1, 3, 7, and 11 are from healthy people for the control experiment. Sample Nos. 2, 4, 5, 6, 8, 9, 10, 12, and 13 are from AMI patients.

In order to verify the reliability of SMC for triple detection of cardiac markers, we use SMC to detect 13 clinical serum samples at a dilution of 500 times and compare them with the results of the ELISA kit used in the hospital (Figures 6A,C,E). The results indicate that the SMC method was consistent with the ELISA method routinely used in hospitals, and four healthy samples (Nos. 1, 3, 7, and 11) and nine patient samples (Nos. 2, 4, 5, 6, 8, 9, 10, 12, and 13) are accurately detected and show a good correlation (cTnI, $R^{2}=0.98729$ ; CK-MB, $R^{2}=0.98859$ ; Myo, $R^{2}=0.98243$ ) (Figures 6B,D,F). It is noted that there is no significant difference in results between the two methods.

In normal human serum, the contents of cTnI, CK-MB, and Myo are below 200 pg/ml, 0.3–4.0, and 12–75 ng/ml, respectively. It is worth noting that the contents of CK-MB and Myo in serum are in the normal range for AMI positive sample 5. The SMC-CL method acquires a content of 2.79 ng/ml CK-MB and 42.6 ng/ml Myo, whereas ELISA obtains a content of 2.8 ng/ml CK-MB and 44.6 ng/ml Myo. In contrast, the result of cTnI shows a positive result for both SMC-CL and ELISA methods, which acquires a content of 242 pg/ml for the SMC-CL method and 264 pg/ml for the ELISA method. It means that the indicator of cTnI is more accurate than that of CK-MB and Myo for sample 5. There are two reasons accounted for this phenomenon. First, as an early indicator of AMI with the characteristics of early rise and fast disappearance, Myo is released into the blood after 1 h of the AMI onset and will decline at 12–24 h. When we do blood sampling after 12 h of AMI onset, Myo levels might have dropped below the diagnostic threshold. Second, when micromyocardial infarction occurs, there is not much damage in the area of the myocardial cell. The contents of CK-MB and Myo are increased above the normal level but do not exceed the diagnostic threshold. Fortunately, AMI can be confirmed by the elevation of cTnI and other clinical symptoms. Since a single indicator detecting cardiac biomarkers by the conventional method (ELISA KIT) cannot give an accurate and prompt diagnosis for AMI, multiplex detecting cardiac markers becomes crucial and essential. The SMC method we developed can detect three cardiac biomarkers simultaneously in 17 min, which meets the need of the clinical application for POCT.

### 3.7 Comparison of Detection Capabilities

To verify the advantages of SMC, we compare the colorimetric method, immunofluorescence analysis method, and surface-enhanced Raman scattering (SERS) method in terms of the number of markers, LOD value, and detection time (Table 1). It shows that SMC has higher sensitivity, a lower operating technology threshold, more portable detection equipment, and, most importantly, a significant advantage in detection time under the premise of simultaneous detection of multiple markers.

**TABLE 1:** Comparison of SMC detection capabilities.

Detection method	CL	CL	Colorimetric	Fluorescence	SERS
Multiplex testing	Y	Y	N	Y	Y
Detection time	17 min	>1 h	1.5 h	>4.5 h	30 min
Performance of operations	Simple	Complicated	Complicated	Complicated	Simple
LOD of cTnI	1.02 pg/ml	6 pg/ml	4.4 pg/ml	2 ng/ml	0.44 pg/ml
CK-MB	1.37 pg/ml	67 pg/ml	300 pg/ml	3 ng/ml	0.55 pg/ml
Myo	4.15 pg/ml	100 pg/ml	270 pg/ml	5 ng/ml	3.2 pg/ml
References	This Work	Zhao et al. (2019)	ELISA KIT	Caulum et al. (2007)	Zhang et al. (2018)

## 4 Conclusion

In conclusion, we developed an SMC for the multiplex detection of the cardiac markers cTnI, CK-MB, and Myo to carry out early and rapid screening of AMI. Manufacturing with 3D printing has the advantages of good reliability, a low production threshold, and diverse individualized design. The time consumption is significantly reduced because antigens and detection antibodies are premixed in the chip. Compared with other methods, it has the advantages of simultaneously detecting multianalytes with higher sensitivity and a faster speed. We believe that the SMC has a great clinical application value in the combined detection of cardiac markers and other biomarkers and the potential in POCT such as emergency vehicles, communities, and families.

## Data Availability

The original contributions presented in the study are included in the article/Supplementary Material; further inquiries can be directed to the corresponding authors.

## References

[B1] Caulum M. M. Murphy B. M. Ramsay L. M. Henry C. S. (2007). Detection of Cardiac Biomarkers Using Micellar Electrokinetic Chromatography and a Cleavable Tag Immunoassay. Anal. Chem. 79 (14), 5249–5256. 10.1021/ac070452v 17566984

[B2] Chan H. N. Tan M. J. A. Wu H. (2017). Point-of-care Testing: Applications of 3D Printing. Lab. Chip 17 (16), 2713–2739. 10.1039/c7lc00397h 28702608

[B3] Chen Y. Yin B. Dong M. Xianyu Y. Jiang X. (2018). Versatile T1-Based Chemical Analysis Platform Using Fe3+/Fe2+ Interconversion. Anal. Chem. 90 (2), 1234–1240. 10.1021/acs.analchem.7b03961 29271635

[B4] Davis T. M. E. Fortun P. Mulder J. Davis W. A. Bruce D. G. (2004). Silent Myocardial Infarction and its Prognosis in a Community-Based Cohort of Type 2 Diabetic Patients: the Fremantle Diabetes Study. Diabetologia 47 (3), 395–399. 10.1007/s00125-004-1344-4 14963648

[B5] Dincer C. Bruch R. Kling A. Dittrich P. S. Urban G. A. (2017). Multiplexed Point-of-Care Testing - xPOCT. Trends Biotechnol. 35 (8), 728–742. 10.1016/j.tibtech.2017.03.013 28456344 PMC5538621

[B6] Dong R. Liu Y. Mou L. Deng J. Jiang X. (2019). Microfluidics‐Based Biomaterials and Biodevices. Adv. Mater. 31 (45), 0–17. 10.1002/adma.201805033 30345586

[B7] Hiramoto K. Ino K. Nashimoto Y. Ito K. Shiku H. (2019). Electric and Electrochemical Microfluidic Devices for Cell Analysis. Front. Chem. 7, 0–8. 10.3389/fchem.2019.00396 PMC6557978 31214576

[B8] Hofmann R. James S. K. Jernberg T. Lindahl B. Erlinge D. Witt N. (2017). Oxygen Therapy in Suspected Acute Myocardial Infarction. N. Engl. J. Med. 377 (13), 1240–1249. 10.1056/NEJMoa1706222 28844200

[B9] Holmvang L. Jurlander B. Rasmussen C. Thiis J. J. Grande P. Clemmensen P. (2002). Use of Biochemical Markers of Infarction for Diagnosing Perioperative Myocardial Infarction and Early Graft Occlusion after Coronary Artery Bypass Surgery. Chest 121 (1), 103–111. 10.1378/chest.121.1.103 11796438

[B10] Hu B. Li J. Mou L. Liu Y. Deng J. Qian W. (2017). An Automated and Portable Microfluidic Chemiluminescence Immunoassay for Quantitative Detection of Biomarkers. Lab. Chip 17 (13), 2225–2234. 10.1039/c7lc00249a 28573279

[B11] Ibanez B. James S. Agewall S. Antunes M. J. Bucciarelli-Ducci C. Bueno H. (2018). 2017 ESC Guidelines for the Management of Acute Myocardial Infarction in Patients Presenting with ST-Segment Elevation: The Task Force for the Management of Acute Myocardial Infarction in Patients Presenting with ST-Segment Elevation of the European Society of Cardiology (ESC). Eur. Heart J. 39 (2), 119–177. 10.1093/eurheartj/ehx393 28886621

[B12] Ji J. Lu W. Zhu Y. Jin H. Yao Y. Zhang H. (2019). Porous Hydrogel-Encapsulated Photonic Barcodes for Multiplex Detection of Cardiovascular Biomarkers. ACS Sens. 4 (5), 1384–1390. 10.1021/acssensors.9b00352 30985109

[B13] Keller T. Zeller T. Peetz D. Tzikas S. Roth A. Czyz E. (2009). Sensitive Troponin I Assay in Early Diagnosis of Acute Myocardial Infarction. N. Engl. J. Med. 361 (9), 868–877. 10.1056/NEJMoa0903515 19710485

[B14] Kim W.-J. Kim B. K. Kim A. Huh C. Ah C. S. Kim K.-H. (2010). Response to Cardiac Markers in Human Serum Analyzed by Guided-Mode Resonance Biosensor. Anal. Chem. 82 (23), 9686–9693. 10.1021/ac101716p 21049960

[B15] Landesberg G. Shatz V. Akopnik I. Wolf Y. G. Mayer M. Berlatzky Y. (2003). Association of Cardiac Troponin, CK-MB, and Postoperative Myocardial Ischemia with Long-Term Survival after Major Vascular Surgery. J. Am. Coll. Cardiol. 42 (9), 1547–1554. 10.1016/j.jacc.2003.05.001 14607436

[B16] Lee C.-Y. Fu L.-M. (2018). Recent Advances and Applications of Micromixers. Sensors Actuators B: Chem. 259, 677–702. 10.1016/j.snb.2017.12.034

[B17] Lim W. Y. Goh C.-H. Thevarajah T. M. Goh B. T. Khor S. M. (2020). Using SERS-Based Microfluidic Paper-Based Device (μPAD) for Calibration-free Quantitative Measurement of AMI Cardiac Biomarkers. Biosens. Bioelectron. 147, 0–10. 10.1016/j.bios.2019.111792 31678828

[B18] Liu J. Geng Z. Fan Z. Liu J. Chen H. (2019). Point-of-care Testing Based on Smartphone: The Current State-Of-The-Art (2017-2018). Biosens. Bioelectron. 132, 17–37. 10.1016/j.bios.2019.01.068 30851493

[B19] McCord J. Nowak R. M. McCullough P. A. Foreback C. Borzak S. Tokarski G. (2001). Ninety-minute Exclusion of Acute Myocardial Infarction by Use of Quantitative point-of-care Testing of Myoglobin and Troponin I. Circulation 104 (13), 1483–1488. 10.1161/hc3801.096336 11571240

[B20] Ohman E. M. Armstrong P. W. Christenson R. H. Granger C. B. Katus H. A. Hamm C. W. (1996). Cardiac Troponin T Levels for Risk Stratification in Acute Myocardial Ischemia. N. Engl. J. Med. 335 (18), 1333–1342. 10.1056/nejm199610313351801 8857016

[B21] Pu J. Ding S. Ge H. Han Y. Guo J. Lin R. (2017). Efficacy and Safety of a Pharmaco-Invasive Strategy with Half-Dose Alteplase versus Primary Angioplasty in ST-Segment-Elevation Myocardial Infarction. Circulation 136 (16), 1462–1473. 10.1161/circulationaha.117.030582 28844990

[B22] Raza W. Hossain S. Kim K.-Y. (2018). Effective Mixing in a Short Serpentine Split-And-Recombination Micromixer. Sensors Actuators B: Chem. 258, 381–392. 10.1016/j.snb.2017.11.135

[B23] Soares R. R. G. Santos D. R. Chu V. Azevedo A. M. Aires-Barros M. R. Conde J. P. (2017). A point-of-use Microfluidic Device with Integrated Photodetector Array for Immunoassay Multiplexing: Detection of a Panel of Mycotoxins in Multiple Samples. Biosens. Bioelectron. 87, 823–831. 10.1016/j.bios.2016.09.041 27657844

[B24] Terray A. Oakey J. Marr D. W. M. (2002). Microfluidic Control Using Colloidal Devices. Science 296 (5574), 1841–1844. 10.1126/science.1072133 12052952

[B25] Thygesen K. Alpert J. S. Jaffe A. S. Simoons M. L. Chaitman B. R. White H. D. (2012). Third Universal Definition of Myocardial Infarction. Circulation 126 (16), 2020–2035. 10.1161/CIR.0b013e31826e1058 22923432

[B26] Viktorov V. Mahmud M. R. Visconte C. (2016). Numerical Study of Fluid Mixing at Different Inlet Flow-Rate Ratios in Tear-Drop and Chain Micromixers Compared to a New H-C Passive Micromixer. Eng. Appl. Comput. Fluid Mech. 10 (1), 182–192. 10.1080/19942060.2016.1140075

[B27] Xie M. Zhang Z. Guan W. Zhou W. Lu C. (2019). Micelle-Mediated Chemiluminescence as an Indicator for Micellar Transitions. Anal. Chem. 91 (4), 2652–2658. 10.1021/acs.analchem.8b03774 30665297

[B28] Yang Y. Chen Y. Tang H. Zong N. Jiang X. (2020). Microfluidics for Biomedical Analysis. Small Methods 4 (4), 0–29. 10.1002/smtd.201900451

[B29] Yin B. Qian C. Wang S. Wan X. Zhou T. (2021). A Microfluidic Chip-Based MRS Immunosensor for Biomarker Detection via Enzyme-Mediated Nanoparticle Assembly. Front. Chem. 9, 0–10. 10.3389/fchem.2021.688442 PMC8193930 34124008

[B30] Yin B. Wang Y. Dong M. Wu J. Ran B. Xie M. (2016). One-step Multiplexed Detection of Foodborne Pathogens: Combining a Quantum Dot-Mediated Reverse Assaying Strategy and Magnetic Separation. Biosens. Bioelectron. 86, 996–1002. 10.1016/j.bios.2016.07.106 27498327

[B31] Yin B. Zheng W. Dong M. Yu W. Chen Y. Joo S. W. (2017). An Enzyme-Mediated Competitive Colorimetric Sensor Based on Au@Ag Bimetallic Nanoparticles for Highly Sensitive Detection of Disease Biomarkers. Analyst 142 (16), 2954–2960. 10.1039/c7an00779e 28725884

[B32] Zhang D. Huang L. Liu B. Ni H. Sun L. Su E. (2018). Quantitative and Ultrasensitive Detection of Multiplex Cardiac Biomarkers in Lateral Flow Assay with Core-Shell SERS Nanotags. Biosens. Bioelectron. 106, 204–211. 10.1016/j.bios.2018.01.062 29428590

[B33] Zhao K. Tang M. Wang H. Zhou Z. Wu Y. Liu S. (2019). Simultaneous Detection of Three Biomarkers Related to Acute Myocardial Infarction Based on Immunosensing Biochip. Biosens. Bioelectron. 126, 767–772. 10.1016/j.bios.2018.11.044 30554098

