# Supplementary material for: Point-of-Care Testing for Multiple Cardiac Markers Based on a Snail-Shaped Microfluidic Chip
Source: Front Chem. 2021 Oct 4;9:741058. doi: 10.3389/fchem.2021.741058 (PMC8521045; doi:10.3389/fchem.2021.741058)
Supplement: Supplementary file 1 [file Table1.DOCX]

Supplementary Material

**Point-of-Care Testing of Multiple Cardiac Markers Based on Snail-Shaped Microfluidic Chip**

Binfeng Yin^1*^, Xinhua Wan^1^, Changcheng Qian^1^, A S M Muhtasim Fuad Sohan^1^, Songbai Wang^2^*, Teng Zhou^3^

^1^School of Mechanical Engineering, Yangzhou University, Yangzhou 225127, China

^2^School of Chemistry and Chemical Engineering, Shanxi University, Taiyuan 030006, China

^3^Mechanical and Electrical Engineering College, Hainan University, Haikou 570228, China

^*^ Corresponding author


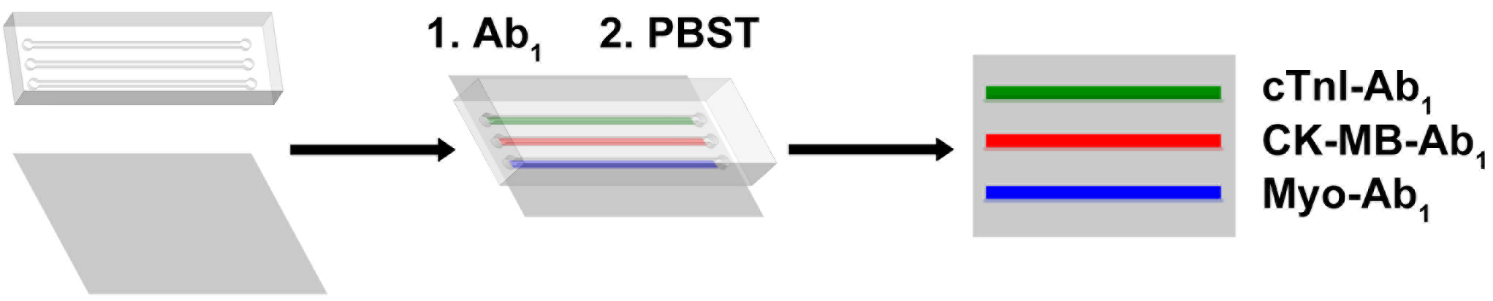


**Figure S1.** The process of coating capture antibodies on a silicone film.

**Conditions of simulating in COMSOL**

The coupled system contains Navier–Stokes equations describing the flow field and a convection-diffusion equation describing the concentration field. Assuming that the fluids in microchannel are incompressible Newtonian fluids, Navier–Stokes equations can be expressed in equations below.

$\rho$ is the fluid density, $\overrightarrow{V}$ is the flow velocity, $P$ is the pressure, $\mu$ is dynamic viscosity. Herein, $\rho=1000 \text{kg}/m^{3}$, $\overrightarrow{V}=0.01 m/s$, .

Convection-diffusion equation can be expressed in equation below.

$c$ is the concentration, $D$ is the fluid diffusion coefficient. Herein, the concentration of the two solutions at the inlet is set at 0 mol/L and 1 mol/L. $D=10^{\text{-12}}\text{m}^{2}\text{/s}$.

We evaluate the mixing efficiency using the following equation.

$$\sigma=1-\sqrt{\frac{\int_{\Gamma} \left(c-\overline{c}\right)^{2}}{A*\overline{c}*2}}$$

$\overline{c}$ is the average concentration, $\Gamma$ is the concentration we measured from the outlet plane, $A$ is the area of the outlet plane.


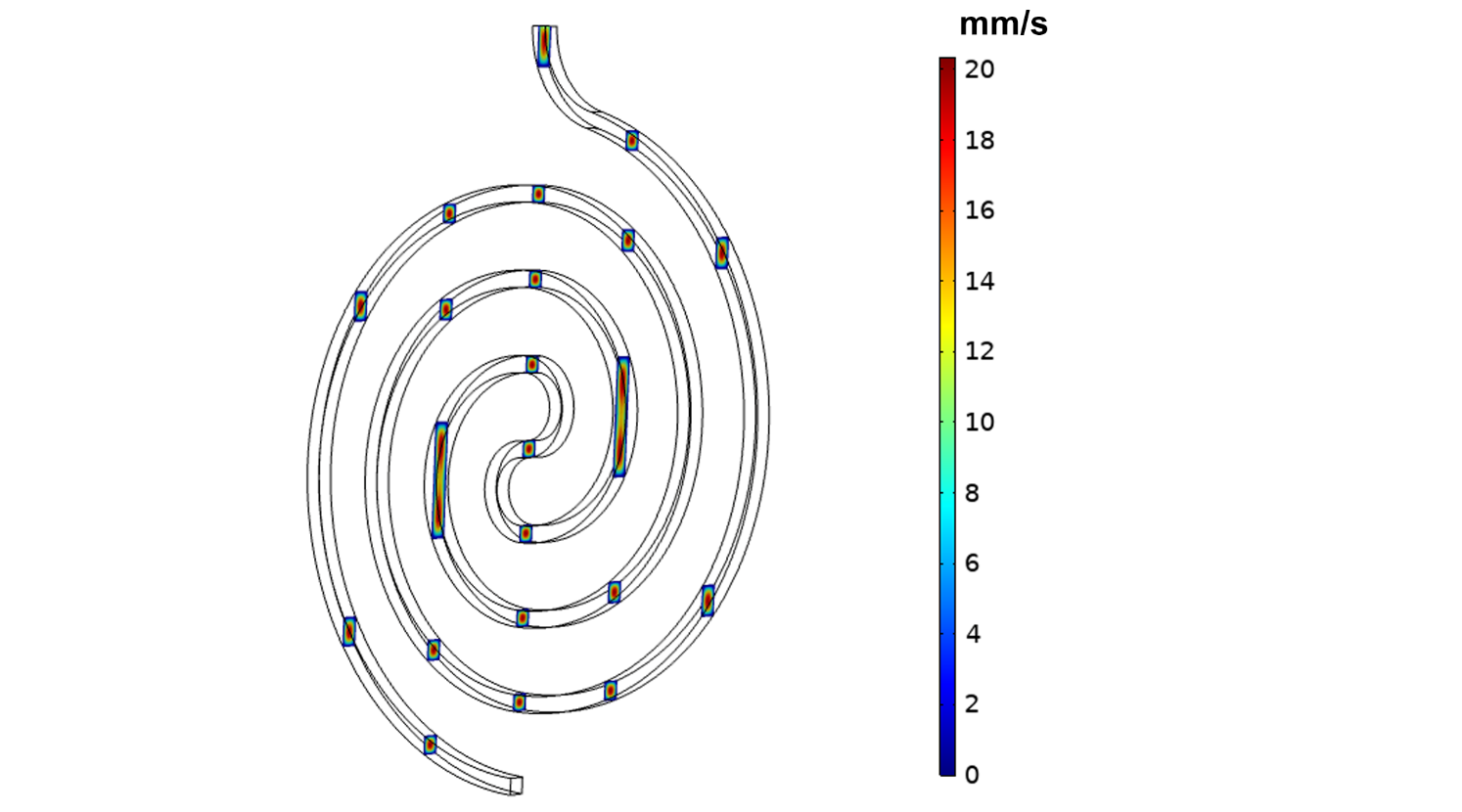


**Figure S2.** Results of simulating flow rate. The velocity of the flow in the SMC is stable via the control of the negative pressure peristaltic pump.


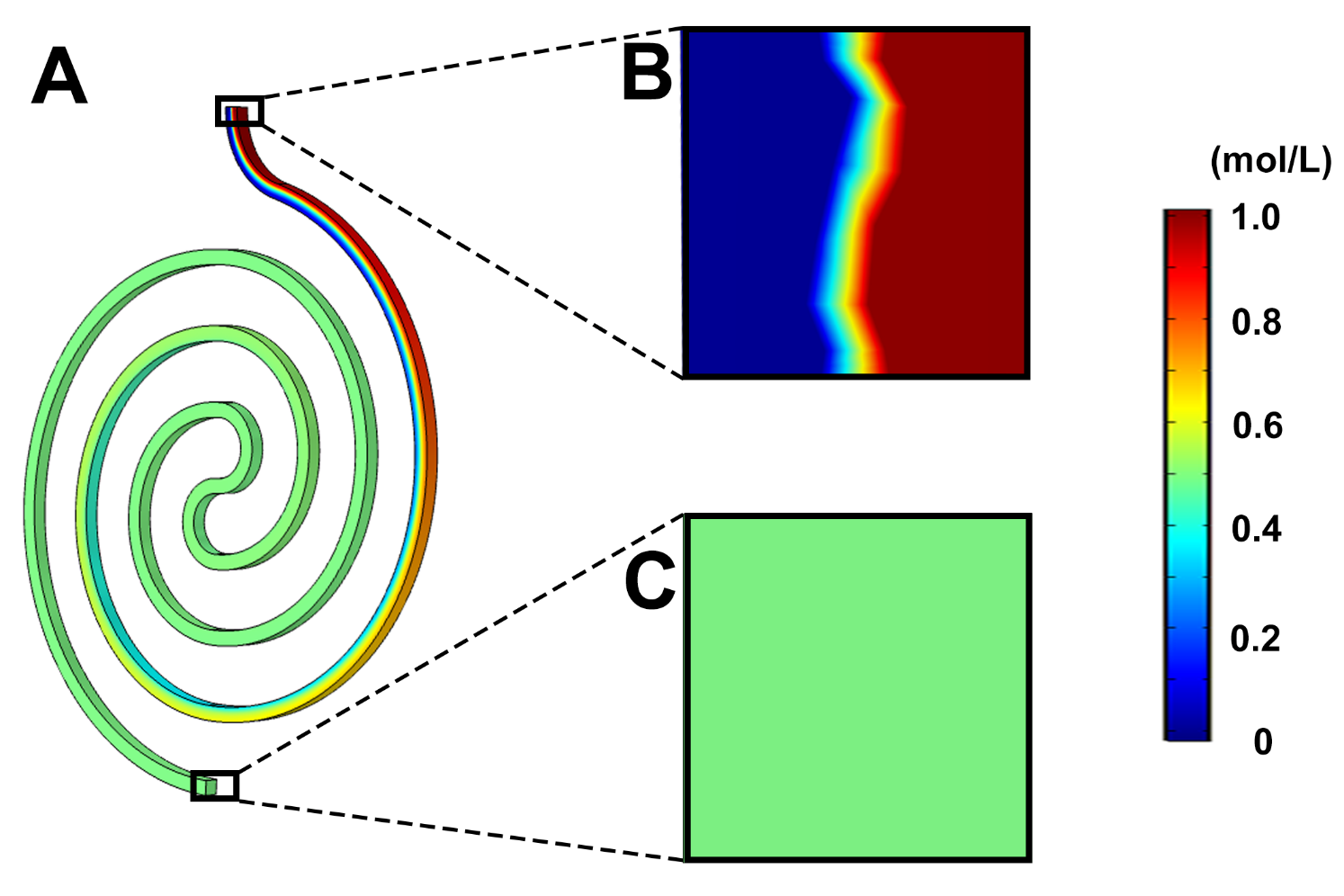


**Figure S3.** Results of simulation about mixing effect by COMSOL software. (A) Distribution of concentration in SMC. (B) The concentration of the two solutions at the inlet is set at 0 mol/L and 1.0 mol/L, respectively. (C) The mixing efficiency at the outlet of Archimedean spiral microchannel is 0.9999. It means the two solutions have been completely mixed.





**Figure S4.** Optimization of concentration of capture antibodies on silicon film. During the experiment, both the solution of antigens and the detection antibodies are 10 μL in volume. The concentrations of all three antigens are 10.24 ng/mL. The concentrations of all three detection antibodies are 3 μg/mL.
